# Design of novel super wide band antenna close to the fundamental dimension limit theory

**DOI:** 10.1038/s41598-020-73478-2

**Published:** 2020-10-01

**Authors:** Shuvashis Dey, Nemai Chandra Karmakar

**Affiliations:** Department of Electrical and Computer Systems Engineering, Monash University, Clayton, VIC 3800 Australia

**Keywords:** Engineering, Electrical and electronic engineering

## Abstract

This paper investigates the design and practical implementation of a Super Wide Band (SWB) antenna along with the application of fundamental bandwidth limitation theory of small antennas in the proposed design. The antenna is designed on a material with permittivity, ε_r_ = 3 where the patch metallization height is maintained as 0.035 mm. The designed antenna is then modified by enhancing the copper patch with an additional layer of 28.5 mm thickness. The proposed antenna achieves a huge frequency range with a ratio bandwidth starting from 96.96:1 to as high as 115.10: 1. The designed antenna operating band with thinner height starts from 1.65 to 160 GHz while with the added patch metallic height, the antenna operates from a minimum of 1.39 to 160 GHz with an average nominal bandwidth of more than 158 GHz. By enhancing the patch height, the antenna spherical volume is utilized more efficiently. Using this principle, the antenna impedance bandwidth is augmented while a reduction in electrical size is achieved. A comparison with the fundamental theories by Chu and Mclean illustrates that the designed SWB antenna electrical size exceeds Mclean and nearly touches the Chu fundamental limit curve. This eventually offers the maximized bandwidth with the most compact size for an SWB antenna. The designed antenna with thinner patch metallization height is practically fabricated and measured up to 67 GHz using Vector Network Analyzer to provide experimental validation.

## Introduction

Ultra-Wide Band (UWB) radio technology has the ability to provide an exceedingly high data rate wireless communication over a short distance. Wireless personal area network (WPAN) based applications have a great demand for ultra-wideband (UWB) frequency band. However, there has been a recent trend to utilize the super wideband (SWB) range which can offer a pervasive service by covering both short and long-range data transmission^1^. Super wide band technology is far more advantageous than the narrow band, and it contains all the advanced characteristics of UWB. Moreover, SWB offers an increased channel capacity, greater time-accuracy and a superior resolution in comparison to that of the UWB^2^.

The most widely used definitions of bandwidth in the antenna community are the ratio and the percent bandwidth. Considering BW as nominal bandwidth which is the difference between the maximum (*f*_H_) and minimum (*f*_L_) frequencies at -10 dB, the ratio bandwidth can be described as: *BR* = *BW*/*f*_L_. Defining the ratio as R = *f*_H_/*f*_L,_ the ratio bandwidth can also be expressed as *BR* = R: 1. SWB indicates a ratio bandwidth equals to or higher than 10:1 which means a larger frequency range compared to the decade bandwidth^3^.

Antenna is an essential part of communication systems, and it is a fundamental element of UWB and SWB radio technology. Advancement in electronic and communication technologies has facilitated the development of miniaturized, efficient and smart antenna systems^4,5^. Antenna miniaturization is a substantial and fascinating topic in the fields related to the electromagnetic and microwave engineering. Miniaturized and versatile antennas have an escalating demand, ever since the introduction of radio frequency (RF) based wireless communications. The requirement of an increased number of multi-functional systems in today’s contemporary world boosts the demand for compact portable terminals further. Such miniature devices involving mobile phones, global positioning system (GPS) systems and radio frequency identification (RFID) equipment necessitate efficient antennas that are small in size. These systems and continuing development of wireless gadgets will keep challenging the community to produce smaller and smarter multi-functional antennas on a persistent basis^6,7^. Especially, the UWB and SWB based applications pose a significant challenge due to the exceedingly stringent regulations about efficiency, size and bandwidth. In the end, such regulations are governed by some specific theoretical factors. Attaining these fundamental theoretical limits is of extreme importance in today’s competitive world, considering the increasing demand for compact antennas with large bandwidth so as to enable an extensive functionality in modern wireless appliances^8^.

This paper introduces the design and study of a novel super wide band antenna having a bandwidth ratio of up to 115.10:1. The proposed design incorporates a monopole antenna and it has a compact and planar structure. The antenna is designed on a commercially available substrate material named Rogers R03003 laminate. The thickness of the copper (metallic layer) patch of the antenna is initially set as 0.035 mm. The operating frequency of this antenna ranges from 1.65 to 160 GHz having a ratio bandwidth of about 96.96:1. The patch metallization height of the designed SWB antenna is then increased to 28.5 mm and its subsequent performance in terms of electrical size and bandwidth is investigated. This patch height is obtained through parametric analysis for the most optimized value of return loss vs. bandwidth. The operating frequency range of the antenna with increased patch height (1.39–160 GHz with a bandwidth ratio of 115.10:1) demonstrates substantial improvement over the antenna with thinner patch metallic layer. The proposed antennas with both patch thicknesses could be termed to have Super Wide Band (SWB) range since they possess an average impedance bandwidth of larger than 158 GHz and exceed the ratio bandwidth, R: 1 = 10:1 by a huge margin. The increment in patch thickness ensures the effective utilization of the antenna spherical volume. This improves the antenna impedance bandwidth which in turn plays a significant role in the electrical size reduction. A comparative study between the designed antennas and classical limitation theories given by Chu and Mclean demonstrates that the analytically obtained antenna electrical size surpasses the Mclean and reaches very close to the Chu fundamental limit curves. This provides an excellent uniqueness to the proposed antenna as it has the maximized achievable bandwidth for the smallest possible size. Here the entire simulation work is carried out using CST Microwave Studio software. The proposed antennas are practically fabricated, and experimental validation is performed through measurement. A vector network analyzer (VNA) having a range of up to 67 GHz is used to measure the fabricated antennas. An in-depth examination of S-parameters and far-field radiation patterns is performed here to indicate the convergence of the simulated and measured results.

The paper is organized as follows: Section “Related works” presents a brief review of the SWB antenna-based papers available in literature while section “Fundamental limitation theory for small antennas” discusses the fundamental limitation theory for small antennas. Section “Antenna design” introduces the design of circular disc monopole based SWB antenna whereas section “Results and analysis” illustrates the analysis of simulated and measured results followed by the comparative study of designed antenna electrical size with respect to the fundamental theories given by Chu and McLean. Finally, the conclusions and future directions are illustrated in section “Conclusions”.

## Related works

In order to attain SWB operating frequency, a range of design methodologies and techniques have been adopted. A closer look in literature reveals an increasing trend of SWB antenna design-based works in recent times^1–3,9–26^. The SWB antenna proposed in^1^ operates in the frequency band 1.44 to 18.8 GHz. It is quite efficient in covering most of the widespread wireless applications including, Bluetooth, LTE, WiMAX along with Digital Video Broadcasting-Handheld (DVB-H) band (1452–1492 MHz) for the portable media player (PMP) applications. However, it is not suitable for high-frequency applications. In^3,9^ the design of different SWB antennas on textile materials are proposed and their conformal characteristics are analyzed. An SWB band of 5–150 GHz is realized in^2,14^. Despite the large frequency span, the antenna cannot be adopted for communications involving S-band or WiMAX due to its inoperability in lower bands. Another antenna ranging from 2.18 to 44.5 GHz is depicted in^15^ which has a miniature size. Nonetheless, many common wireless application bands such as GPS, PCS, DCS and UMTS cannot be covered by using this antenna. It also fails to operate in the Q band (33–50 GHz) frequency range. A significant addition to the ever-increasing list of SWB antennas is depicted in^17^ which covers a huge range starting from 11 to 200 GHz; however, it has the limitation in covering most of the common wireless applications like ISM, WLAN, UWB and so on. A keen investigation on the reported SWB antennas in literature reveals that the antenna proposed in^26^ has debatably the highest achieved bandwidth ratio so far, with a ratio of 63.3:1. Although the proposed antenna in^21^ claims to offer a ratio bandwidth of 111.1:1, however, this antenna does not essentially cover the entire SWB band in an uninterrupted manner, since it is designed to suppress the 4.7–6 GHz^21^. A comprehensive survey on SWB antenna-based papers is illustrated in^27^. It provides a thorough comparative analysis between the reported antennas based on various parameters. The SWB antennas depicted in this paper have widespread operability over a range of popular radio wave based wireless systems including ultra-wide band (UWB), global positioning systems (GPS), WiMAX technology, Long term evolution (LTE), 5G millimeter wave and many others. Besides, the proposed antennas can also operate in the Ku, K, Ka, S, C, X and Q bands which enables them to be used in satellite or global microwave communications and for space radio science studies^28^. These antennas also cover the band of recently introduced IEEE 802.11aj (45 GHz) which would offer a maximum data rate of more than 10 Gbps to satisfy the next round mobile traffic growth^29^.

## Fundamental limitation theory for small antennas

An electrically small antenna can be described in terms of the antenna’s radian length, ℓ = λ/2π and highest dimension, *a.* If the wavenumber of the electromagnetic wave is represented by, k = 2π/λ, a small antenna can be defined to have the following inequality:

$$ \begin{aligned} & a \le \lambda /2\pi \le \, 1/{\text{k}} \\ & {\text{k}}a \le 1 \\ \end{aligned} $$

Put differently, an antenna that fits within a sphere having the radius of *a* = 1/k can be defined as a small antenna. In general, the highest dimension of such an antenna is smaller than λ/4.

Quality factor, ${\text{Q}}$ is an important antenna parameter which establishes a link between antenna electrical size and bandwidth. It can be mathematically defined by the following equation:

$$ {\text{Q}} = \left\{ {\begin{array}{*{20}l} {\frac{{2\omega {\text{W}}_{{\text{e}}} }}{{{\text{P}}_{{{\text{rad}}}} }}} \hfill & {{\text{W}}_{{\text{e}}} > {\text{W}}_{{\text{m}}} } \hfill \\ {\frac{{2\omega {\text{W}}_{{\text{m}}} }}{{{\text{P}}_{{{\text{rad}}}} }}} \hfill & {{\text{W}}_{{\text{m}}} > {\text{W}}_{{\text{e}}} } \hfill \\ \end{array} } \right. $$

Here, W_e_ and W_m_ are the time-average, non-propagating, stored electric and magnetic energy respectively and ω denotes the angular frequency while P_rad_ represents the radiated power. At higher values, the antenna ${\text{Q}}$ is assumed to be reciprocal of its fractional bandwidth. However, at lower values of ${\text{Q}}$, the antenna input impedance undergoes a slow variation with the frequency which results in a broad bandwidth potentiality of the antenna^30^.

1 $$ \begin{aligned} Bandwidth & = \frac{{f_{{{\text{upper}}}} - f_{{{\text{lower}}}} }}{{f_{{{\text{center}}}} }} \\ & = \frac{1}{{\text{Q}}} \\ \end{aligned} $$

For the lowest transverse magnetic (TM) mode, L.J. Chu derived an expression^31^ of ${\text{Q}}$ as follows:

$$ {\text{Q}} = \frac{{1 + 3{\text{k}}^{2} a^{2} }}{{{\text{k}}^{3} a^{3} \left[ {1 + {\text{k}}^{2} a^{2} } \right]}} $$

This differs from that obtained from his equivalent second-order network, which gives-

2 $$ {\text{Q}} = \frac{{1 + 2{\text{k}}^{2} a^{2} }}{{{\text{k}}^{3} a^{3} \left[ {1 + {\text{k}}^{2} a^{2} } \right]}} $$

The two expressions are similar for lower values of k*a* (high ${\text{Q}}$) but begin to differ towards the upper limit of k*a* (i.e. as it approaches 1).

As k*a* <  < 1, the equation becomes:

3 $$ {\text{Q}} \cong \frac{1}{{{\text{k}}^{3} a^{3} }} $$

This relationship implies that the antenna size reduction results in a rapid increment in ${\text{Q}}$ values. Thus, a comparative look at Eqs. (1) and (3) reveals that a reduction in antenna size essentially reduces the bandwidth. Hence, it is a critical challenge for the electrically small antennas to have an optimally increased bandwidth with reduced size.

McLean proposed an alternate technique for calculating the antenna quality factor in^32^. In comparison to Chu’s approximate equation, this technique provides an exact expression for ${\text{Q}}$ calculation. This expression complies with Chu’s theory at higher ${\text{Q}}$ values, however, when the ${\text{Q}}$ is low, the two theories deviate from each other significantly.

Mclean’s Equations^32^ for calculating ${\text{Q}}$ is as follows-

4 $$ {\text{Q}} = \frac{1}{{{\text{k}}^{3} a^{3} }} + \frac{1}{{{\text{k}}a}} $$

To validate the efficacy of fundamental limit theory, it is essential to apply on and compare with practically devised antennas. Such an approach on a range of antennas is reported in the literature and it is inferred that none surpass the fundamental limits. Hence, the classical theories by Chu and McLean are believed to offer realistic limits^33^.

To evaluate the electrical size of broadband small antennas, no universal method has been introduced so far. Classical theories can be effectively utilized for narrow band antennas defined using fractional-bandwidth. However, for ratio or octaval bandwidth which characterizes the broadband antennas, these theories are not very suitable. Nonetheless, here also, the concepts from Chu and McLean theories can be incorporated to obtain the optimum antenna sphere size (electrical size) that gets close to the fundamental limitation theory. In this case, the wideband antenna wavenumber calculation considers the lower-bound frequency of the operating band^34^.

## Antenna design

**Figure 1:**
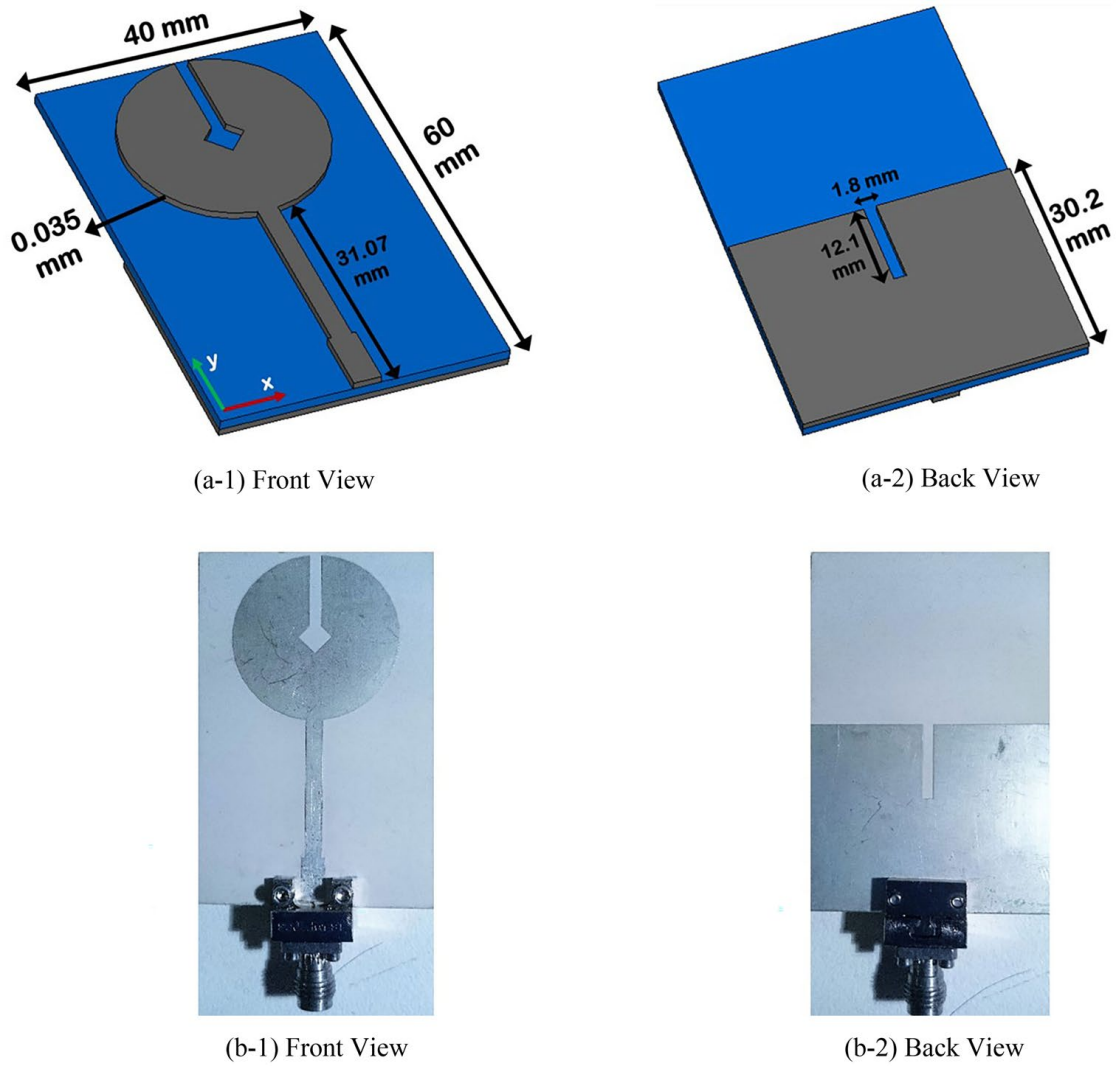
SWB antenna on RO 3003 substrate having patch thickness of 0.035 mm (35 um) (**a**) simulated, (**b**) fabricated.

**Figure 2:**
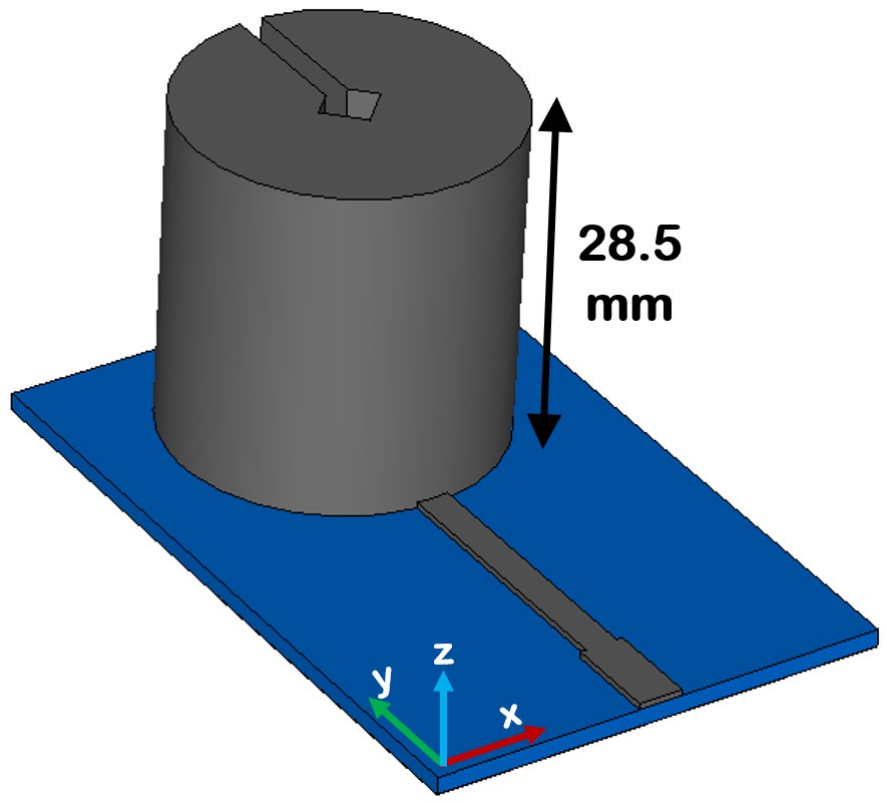
Modified antenna having patch thickness of 28.5 mm.

Here, a circular disc monopole antenna is chosen since it has got a simple structure. The lightweight and low-profile nature of this antenna allow easy integration with substrate materials. With respect to patch height, two different antennas are designed here. The proposed planar antennas with SWB operating band are illustrated with all their dimensions in Figs. 1 and 2. Figure 1 portrays the antenna on substrate Rogers RO 3003 laminate having a relative permittivity, ε_r_ = 3 with a patch metallization height of 35 µm. The dissipation factor of this laminate is tan δ = 0.0013. The designed antenna has a dimension of 60 × 40 mm^2^ and the thickness of the RO 3003 laminate is 1.52 mm. The radius of circular disc (R) for both of the antenna patches is 14 mm which is fed by a 50-Ω microstrip feed line. The circular disc incorporates a square-shaped slot in its center which is added to another rectangular slot having a dimension of 12.13 × 2 mm^2^. Both of the antennas have a 30.2 mm long partial ground plane at the back of their substrate. To enhance the antenna impedance bandwidth, a rectangular notch of 12.1 × 1.8 mm^2^ is introduced on the ground plane as well.

Figure 2 shows the 2nd antenna whose patch height is modified while keeping the rest of the corresponding dimensions intact. The increased patch metallic height is selected as 28.5 mm which is found by using a CST microwave studio based parametric study. This patch thickness increment is instigated to augment the volumetric efficiency by utilizing the complete enclosed antenna spherical volume.

## Results and analysis

The simulated and experimental results of the proposed antennas are analyzed in this section. It presents a detailed study of return loss, radiation patterns and surface currents along with the comparison of S-parameter and gain for antennas with different patch heights. The electrical sizes of both antennas are calculated thereafter; which are then compared with the theoretically obtained Chu and McLean curves representing fundamental limitation theory.

### Simulated return loss, radiation patterns, gain and surface current distribution of antenna with thin patch

**Figure 3:**
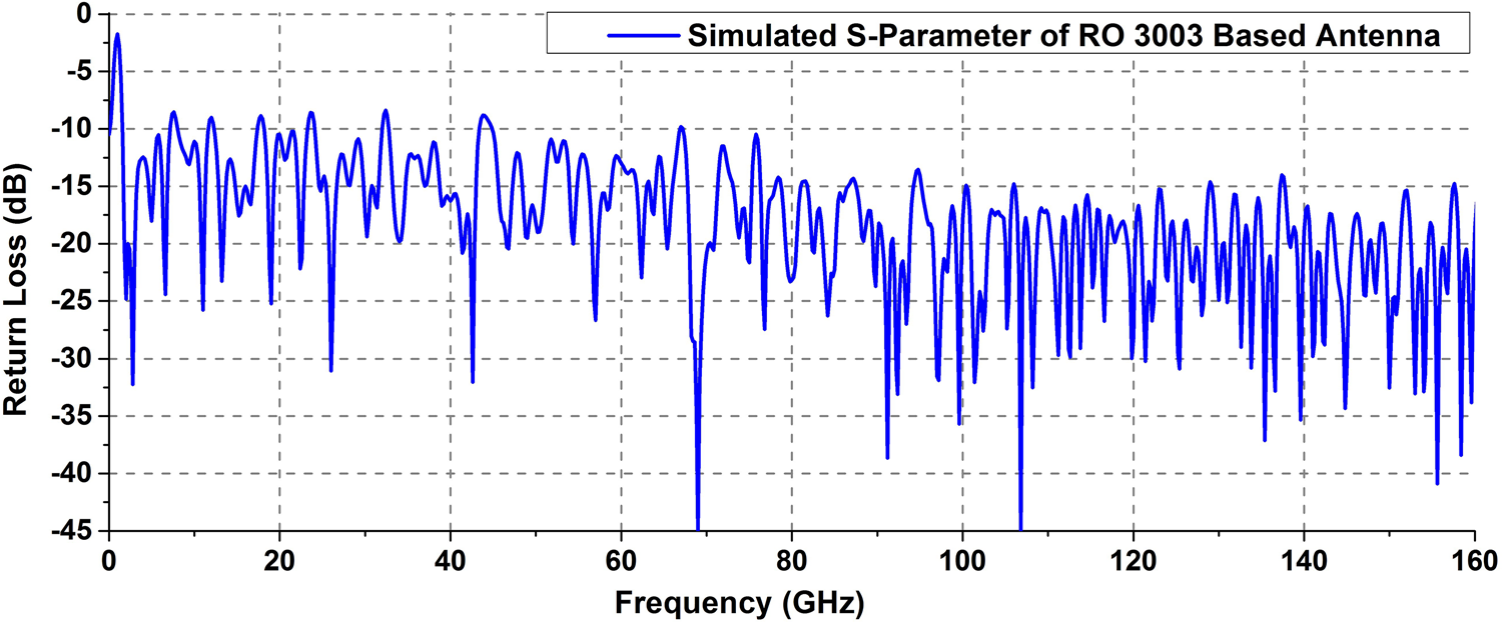
Simulated S-parameter of RO 3003 based antenna.

Figure 3 shows the simulated return loss vs frequency for the antenna with thin patch metallization on RO 3003 substrate. It is evident from Fig. 3 that the designed antenna occupies a frequency range of more than 158 GHz. The proposed antenna operates from 1.65 to 160 GHz with a considerably good impedance matching throughout its operating bandwidth.

**Figure 4:**
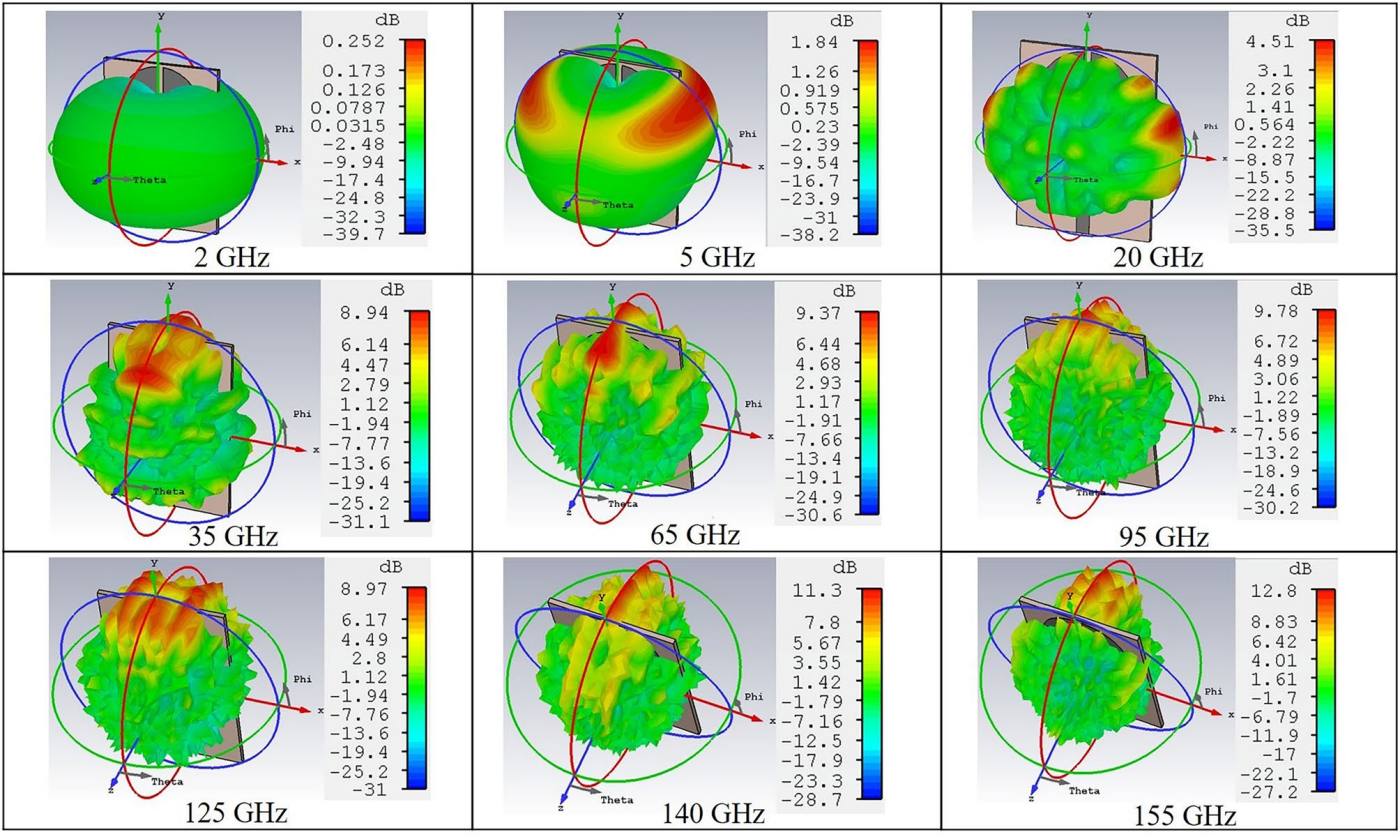
Simulated 3D radiation pattern of the designed antenna.

**Figure 5:**
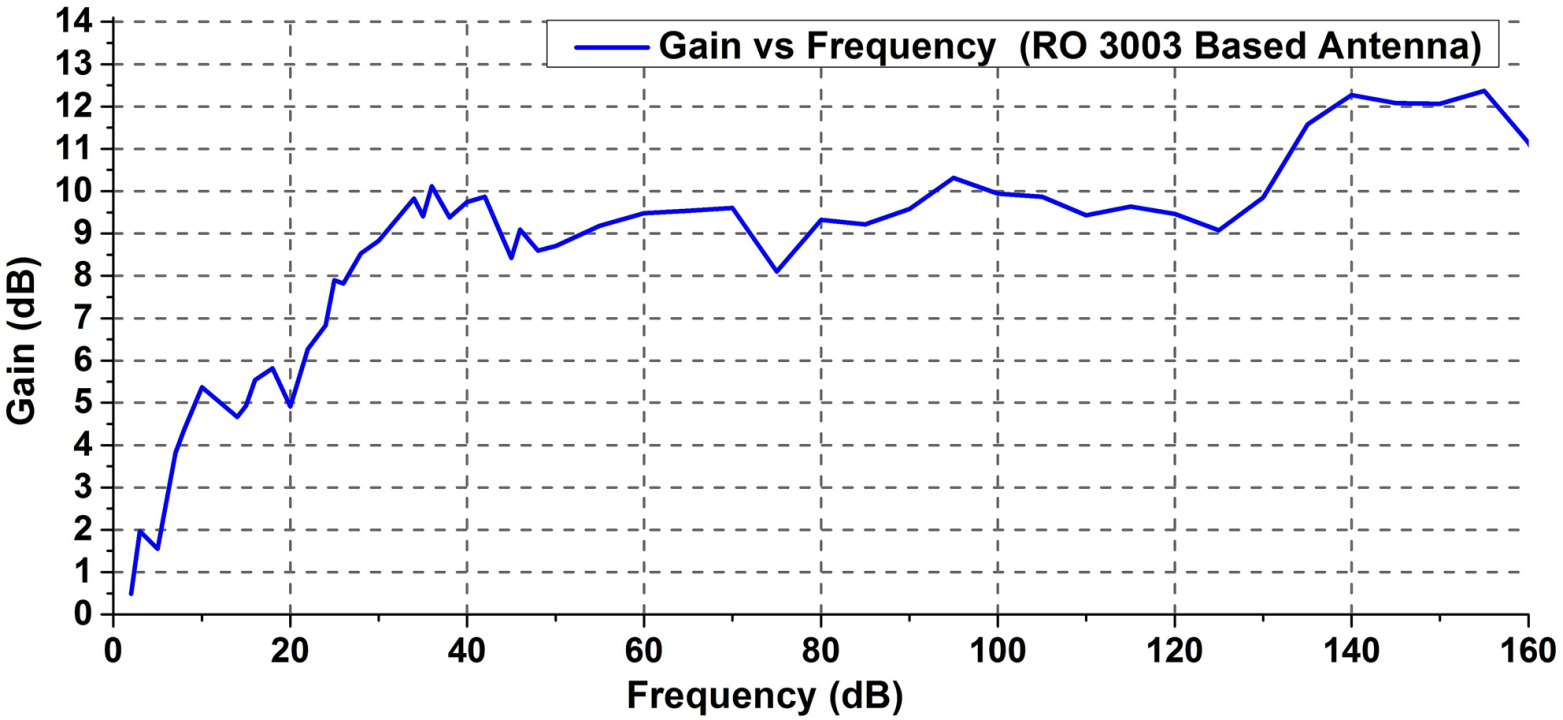
Simulated gain vs frequency of RO 3003 based antenna.

Figures 4 and 5 show the simulated 3D radiation patterns and gain plot of the antenna respectively. The radiation patterns at different frequencies illustrate that at lowers bands, the antenna exhibits an omnidirectional pattern. However, with increasing frequencies, the radiated power mostly gets confined at the top portion of the antenna which results in an almost directional pattern. This, in turn, results in a reduction of antenna 3-dB beamwidth at higher frequency ranges. Except for a few anomalies, the simulated gain vs frequency plot in Fig. 5 depicts an increasing trend of gain with frequency increment. At lower bands, the amplitude of gain appears to be quite low, however, as the frequency gets higher, the gain also increases which reaches to a value of more than 7 dB after 25 GHz. Based on the operating frequency, the efficiency of the designed antenna ranges from a minimum of 64% to a maximum of 93% approximately. This indicates that apart from being a good resonator, the proposed antenna is quite an efficient radiator as well.

From the s-parameter plot depicted in Fig. 3, it can be seen that the first resonance (f_1_) of the proposed antenna occurs at 1.65 GHz. In case of circular disc monopole based Super Wide Band (SWB) antennas, the first resonant frequency (fundamental mode) is determined by using the disc diameter (D) which roughly corresponds to the quarter wavelength at this fundamental frequency^35^. For the disc radius, R and wavelength, λ_1_ at first resonance, the relationship is given by^35^:

5 $$ {\text{D}} = 2{\text{R}} \approx \frac{{\uplambda _{1} }}{4} $$

For the velocity of light, C = 3 × 10^8^ ms^−1^ and dielectric constant, ε_r_, the corresponding wavelength at a given frequency, f can be determined from the following equation:

6 $$\uplambda = \frac{{\text{C}}}{{{\text{f}}\sqrt {\upvarepsilon _{{\text{r}}} } }} $$

**Figure 6:**
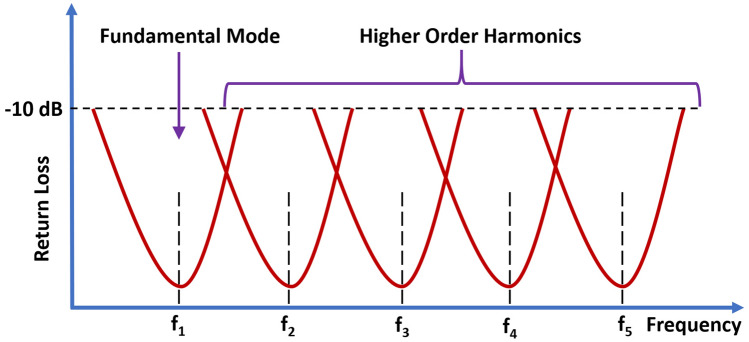
Overlapping of multiple resonance modes to form super wide bandwidth^37^.

By using Eq. (6), for the substrate dielectric constant, ε_r_ = 3, the corresponding wavelength (λ_1_) for the first resonant frequency can be calculated as 104. 97 mm. Accordingly, the quarter wavelength at this frequency is 26.24 mm. As mentioned in section IV, the circular disc radius (R) of the proposed antenna is 14 mm. This essentially gives a disc diameter (D) value (28 mm) close to the quarter wavelength at 1.65 GHz. The circular disc monopole supports multiple resonant modes. The presence of perturbing notches on the disc and the ground plane also introduces additional resonances^33,36^. Once the fundamental frequency is defined based on the disc dimensions, the rest of the resonances can also be obtained since they are essentially the higher order harmonics (modes) of the first resonance. The operational principle of the SWB antenna is shown in Fig. 6. The super wideband spectrum requires the adjacent resonance modes to overlap with each other. Such overlapping of multiple resonance modes closely distributed over the entire frequency spectrum helps the antenna to achieve super wide bandwidth^35,37^.

**Figure 7:**
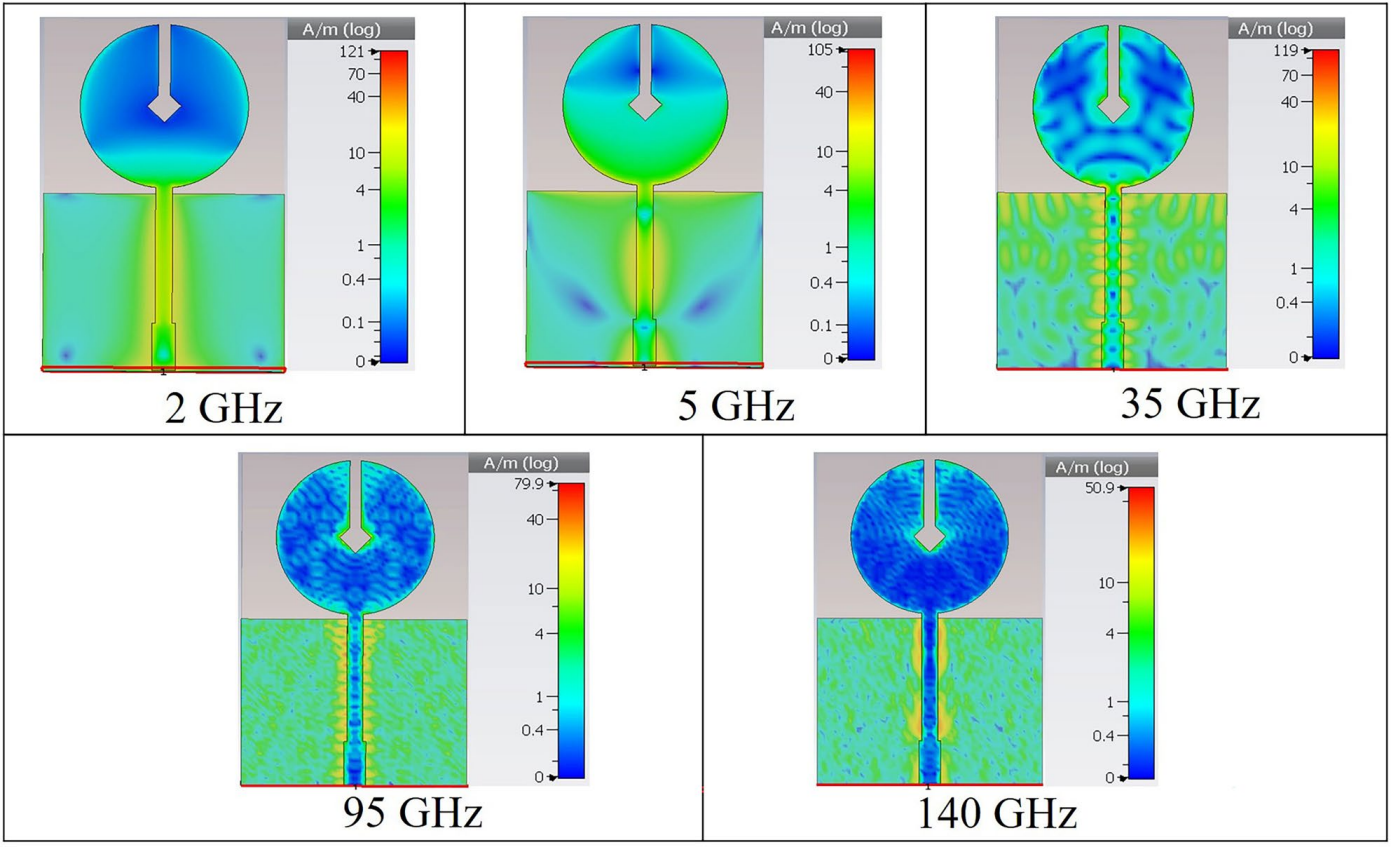
Surface current distribution of antenna at different sample frequencies.

The radiation mechanism of the proposed antenna can be explained from its surface current distribution at different frequencies. Figure 7 shows the current distribution of the antenna at different sample frequencies starting from 2 to 140 GHz. The antenna resonance mode variation is depicted here in terms of the surface current analysis. From Fig. 7, it is evident that the magnitude of current is comparatively higher around the feed line which makes it a better indicator of mode variation.

At 2 GHz (near first resonance), only the fundamental mode operates. In this case, the current oscillates at the top and middle portions of the circular disc which results in a broad envelope hump indicating a standing wave (no net current propagation). The perturbing notch of the disc is also surrounded by the standing wave while a small amount of current travels along the lower disc edge. This causes the corresponding 3D radiation pattern (Fig. 4) to appear as a donut with null radiated power at the top portion of the antenna^35,37^.

At 5 GHz, a slightly distorted yet donut shaped radiation pattern is exhibited. The surface current distribution at this frequency reveals the presence of second harmonic and a standing wave around the rectangular notch of the disc. In this case, the current oscillation is mainly confined at the top portion of the disc and a triangle shaped standing wave pattern is created. Since the rectangular notch is surrounded by the standing wave, there exists a radiating null at the antenna top (similar to the 2 GHz case). However, at this frequency, along with the lower edge, the current also travels in the middle portion and the top two edges of the circular disc. As a result, the antenna radiates with a deformed donut shape having the maximum radiated power at around the top two angular directions, namely 45° and 135°. The surface current analysis and the nearly omnidirectional patterns exhibited at 2 GHz and 5 GHz frequencies indicate that at such lower frequencies, the antenna mainly operates in the standing wave dominating modes^35,37^.

As the frequency increases, the circular disc monopole starts to exhibit higher order harmonics with a hybrid mode of operation which consists of both standing and travelling waves. For example, at 35 GHz, an increased number of harmonics can be observed which results in a complex heterogeneous surface current distribution. At such a high frequency, the travelling wave starts to become predominant although there are scattered areas on the disc where current oscillations occur to form standing waves. Interestingly, unlike the lower frequency cases, here, the perturbing notches of the disc are not surrounded by standing waves. Instead, a significant amount of current travels through that region. This prompts the antenna to confine most of the radiated power to the upward direction which indicates the occurrence of maximum radiation from the specified notch area. Due to the presence of scattered standing wave regions on the circular patch, the antenna encounters radiating nulls at the corresponding locations. This deviates the antenna radiation pattern from being omnidirectional^35,37^. A similar phenomenon can be observed at a much higher frequency of 95 GHz. Here, the number of higher order modes increase to such as extent that they start to overlap. The antenna radiates mostly on the upward direction and due to the increasing presence of combined travelling and standing waves, the far-field pattern incurs further distortion.

At extremely high frequencies such as 140 GHz, the antenna is quite electrically large. An animation of surface current distribution reveals that the antenna has a significantly high number of resonance modes at this frequency which are completely overlapped with each other. A hybrid mode of operation still persists here, while the propagation of travelling waves become more critical. This is because the wavelength of the travelling EM wave is extremely short in comparison to the antenna structure. The surrounding area of the perturbing notch of circular disc experiences the most current flow which causes the antenna to radiate sharply on the upward direction. In this case, the region of travelling current surrounding the notch is shallow in comparison to that of the lower frequency cases such as 35 GHz. Consequently, the area of maximum radiated power is also quite confined which is evident from the corresponding 3D radiation pattern shown in Fig. 4. Due to the presence of a significantly dense combination of travelling and standing wave over the entire disc, the overall radiation pattern is completely distorted with high asymmetry^35,38^.

### Measured return loss, radiation patterns and gain of antenna with thin patch

The proposed antenna with thin patch metallization is practically fabricated and measured by using Agilent programmable network analyzer (PNA) E8361A. The maximum operating frequency of this PNA is 67 GHz. Therefore, the designed antenna performance is measured up to this frequency range.

**Figure 8:**
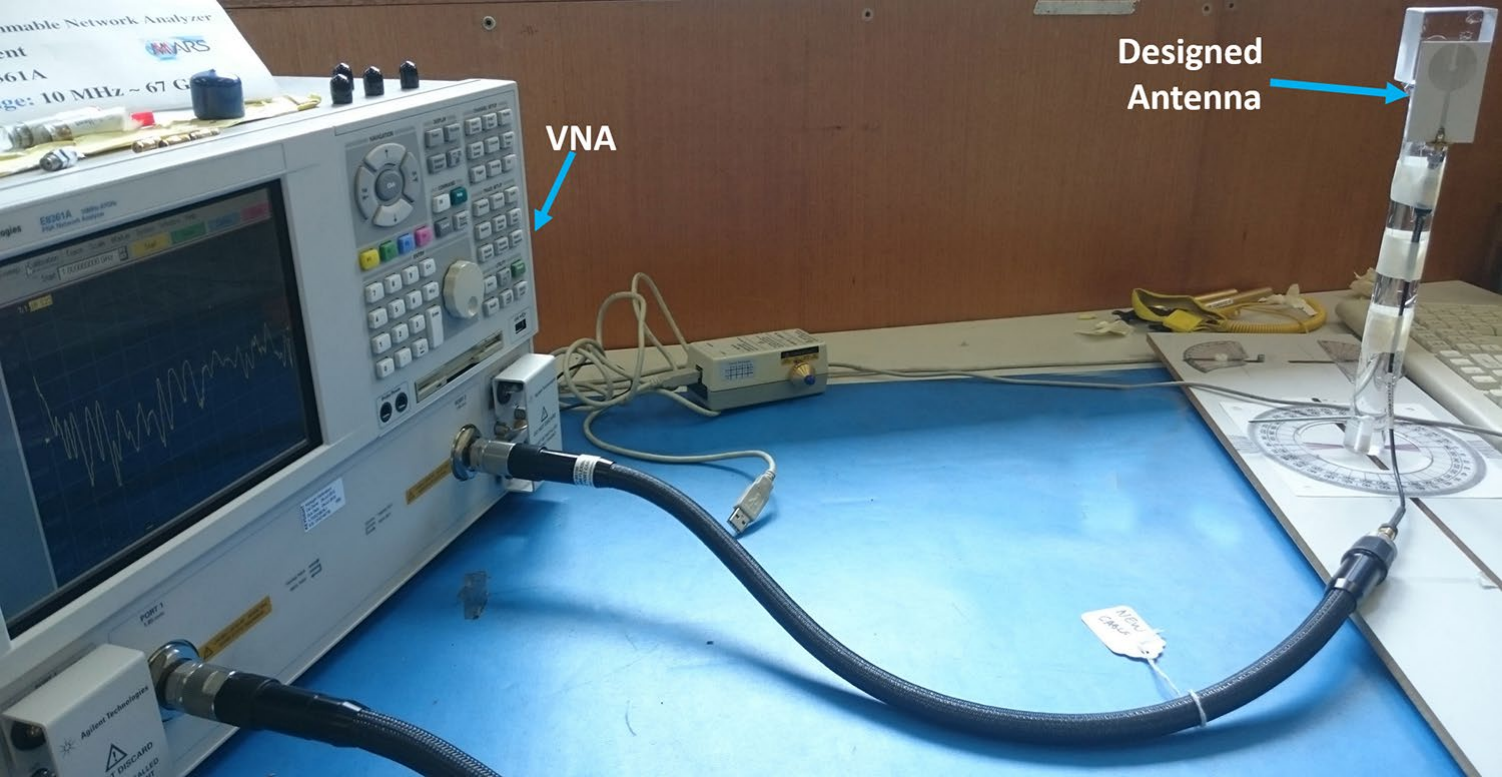
Measurement set-up for return loss calculation of RO 3003 based antenna.

**Figure 9:**
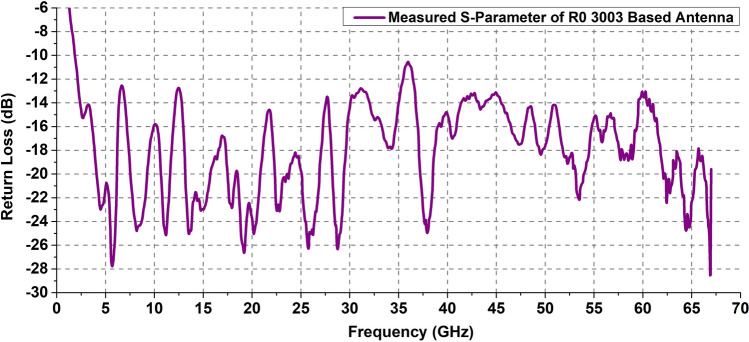
Measured S-parameter of RO 3003 based antenna.

Figures 8 and 9 show the measurement set-up and the measured return loss vs frequency plot for RO 3003 based antenna respectively. It can be observed that the antenna exhibits an excellent impedance matching with a return loss of greater than 10 dB for the entire occupied band of 1.75 to 67 GHz. The slight offset of 100 MHz from the simulated lowest operating frequency band (1.65 GHz) occurs due to fabrication error or connector settings.

**Figure 10:**
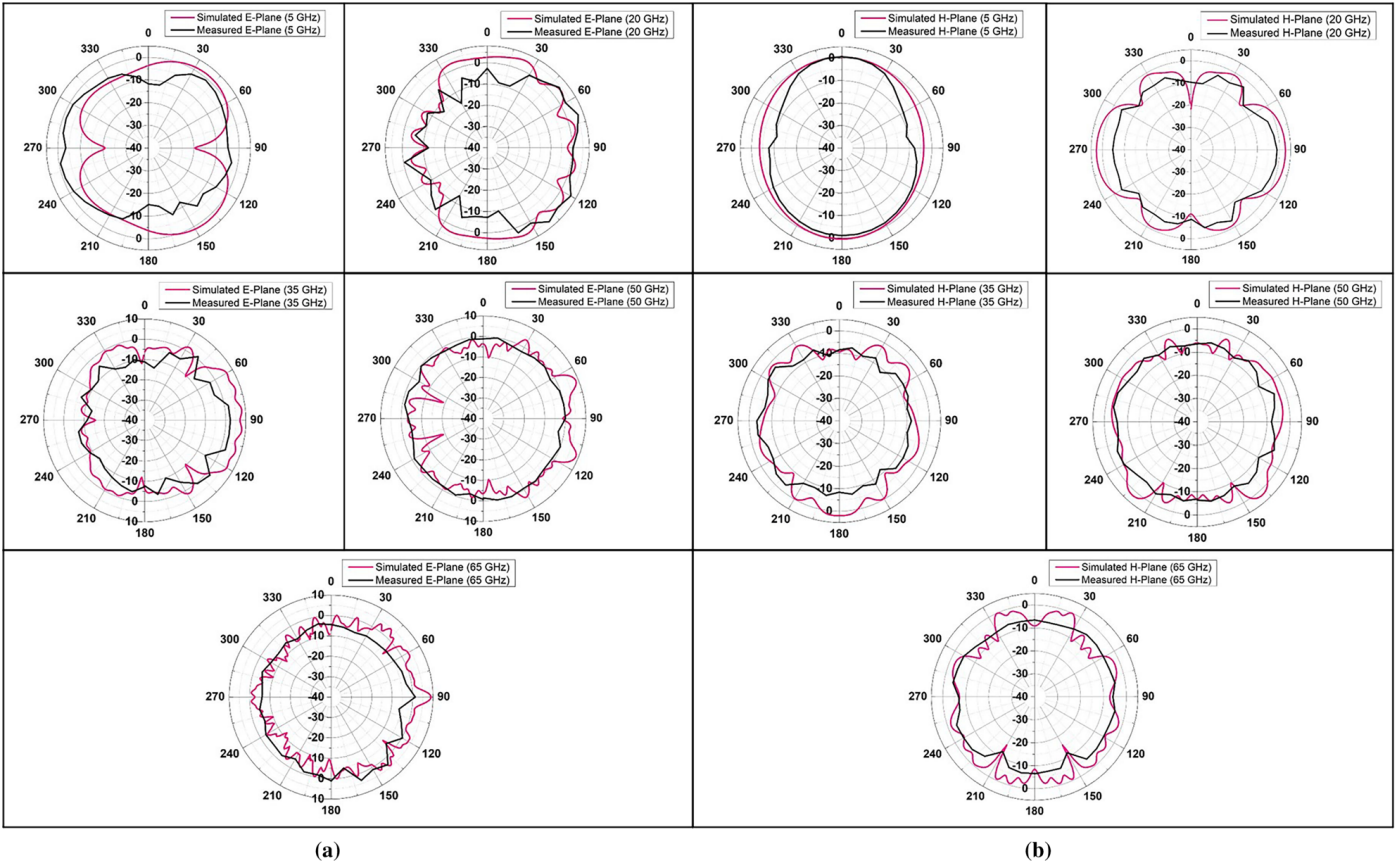
(**a**) E-Plane (**b**) H-Plane Radiation pattern of fabricated SWB antenna.

Figure 10a,b show the comparative figures of simulated and measured E and H plane radiation patterns of the planar SWB antenna on RO 3003 substrate respectively. In this case, the antenna under test and a similar calibrated antenna are placed approximately 160 cm apart in order to accommodate the far-field distance at the highest operating frequency (67 GHz). From the figures, it is evident that the antenna exhibits omnidirectional radiation characteristics at low frequency (5 GHz). The radiation patterns exhibit some distortions at lower bands such as 20 GHz while at higher bands, the patterns deviate from being omnidirectional with a large number of ripples.

**Figure 11:**
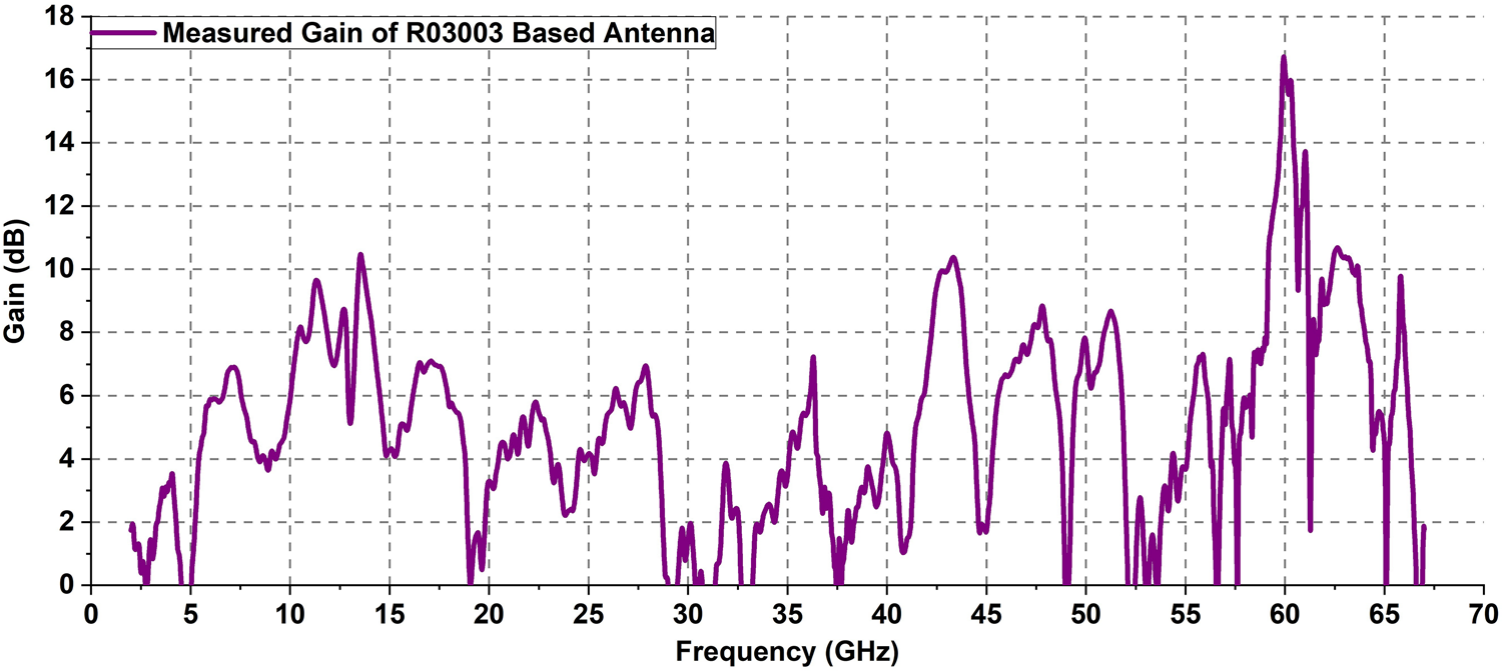
Gain versus frequency plot of the designed antenna.

The measured gain vs frequency plot in Fig. 11 shows that the gain pattern follows the simulated results depicted in Fig. 5. Alike the simulated results, the measured gain also tends to have low magnitude at lower frequency bands. As the frequency gets higher, the gain magnitude tends to increase in general although a significant amount of fluctuation is evident. Interestingly, the peak gain values of the antenna seem to exceed the simulated results though the overall gain values in the entire frequency band are mostly consistent with predicted values. The designed antenna on RO 3003 substrate has a peak gain of 17.19 dB at 60 GHz.

It can be observed that at some specific frequencies the antenna has a measured gain of lower than 0 dB. A significant reason for having such a low gain is the direction at which the gain measurement has been carried out. The designed antenna has a non-omnidirectional radiation pattern at higher frequencies. This means, depending on frequencies, the direction of maximum radiation of the antenna is varied. For example, at frequencies around 35 GHz and above, the radiation is mostly confined to the end-fire (along the antenna axis) direction although some radiated power exists in the broadside (perpendicular to the antenna plane) direction as well. Due to such end-fire radiation, there exist some frequency bands where the radiated power level is very low in the broadside direction. The gain measurement of the proposed antenna is performed in the broadside direction. Therefore, the frequency bands with low radiated power in that direction exhibit a lower gain. However, even at the frequencies where the measured gain is low, the antenna has reasonably high radiation at other directions. From the 3D radiation patterns, it can be seen that antenna often radiates either at both of its top sides (5 GHz case for example) or at the end-fire direction (35 GHz). This indicates that the antenna is efficient enough even at the frequencies where the gain is measured to be lower than 0 dB. The simulated antenna gain depicted in Fig. 5 exhibits the maximum gain (at any direction) over the entire frequency range whereas the measured gain is considered only for a single direction. In addition to the above, the designed antenna is associated with different types of losses including the conduction (ohmic) loss and surface wave loss. These losses also accumulate to affect the overall antenna efficiency and gain at different frequencies.

### Comparison of simulated antenna parameters for different patch heights

**Figure 12:**
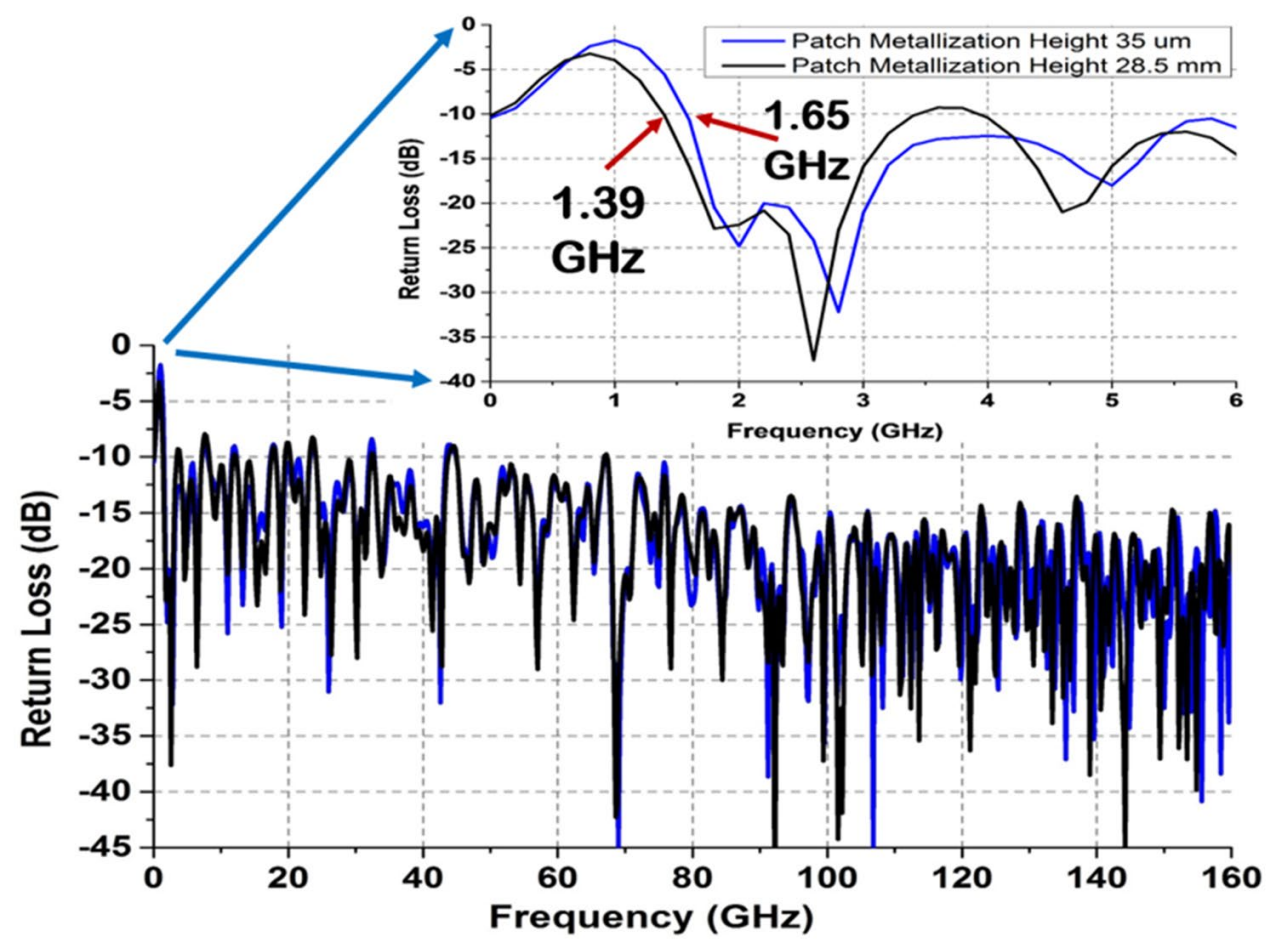
S-parameter comparison of antennas having different patch thicknesses.

**Figure 13:**
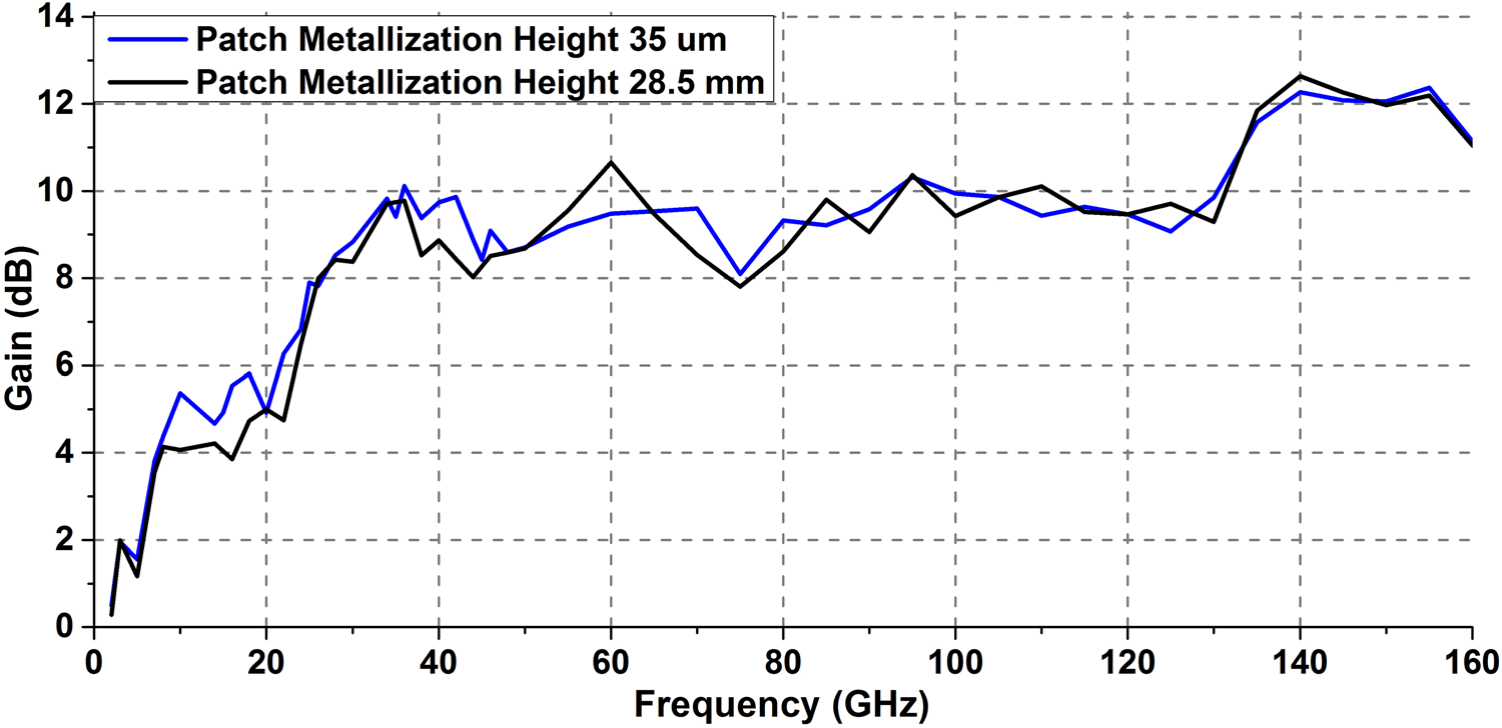
Gain plot comparison for antennas having different patch thicknesses.

Figure 12 illustrates the return loss comparison of designed antennas with different patch metallic thicknesses. The antenna having thick patch (28.5 mm) starts to resonate from 1.39 GHz frequency which is comparatively much lower than that of the thin patch (1.65 GHz). Hence, it is evident that the operating bandwidth of the designed antennas can be improved by enhancing the patch thickness. For the case of the antenna with increased patch height, the nominal bandwidth increases to 158.61 GHz in comparison to 158.35 GHz of the case having thin patch. The gain vs frequency plot comparison depicted in Fig. 13 shows that the increased patch height does not impact much on the gain of the proposed antennas.

The frequency-wise evaluated front-to-back ratios of the proposed antennas are depicted in Table 1. As the radiation pattern is mostly omnidirectional at lower bands, the calculated ratio for antennas with both patch heights are found to be 0 dB at 2 GHz. At higher frequencies, the radiation pattern starts to deform which results in a fluctuation of the front-to-back (F**\**B) ratio.

**Table 1:** Front-to-back ratio of antennas with both patch metallization heights at different frequencies.

Frequency	Front-to-Back Ratio for patch height 0.035 mm		Front-to-Back Ratio for patch height 28.5 mm	
	Linear	dB	Linear	dB
2 GHz	1	0	1	0
15 GHz	5.94	7.74	1.87	2.72
35 GHz	59.56	17.75	78.88	18.97
65 GHz	14.79	11.7	20.27	13.07

### Calculation of SWB antenna electrical size

To evaluate the antenna performance with respect to size and bandwidth, the antenna electrical size for both thin and thick patch heights are calculated. An optimized antenna volumetric efficiency improves the impedance bandwidth while reducing its electrical size. In general, conventional antennas lack in utilizing their spherical volumes in an efficient manner due to which they cannot get close to the fundamental dimension limits specified by Chu and McLean curves. The optimal use of enclosed antenna spherical volume maximizes its impedance bandwidth by reducing the non-radiated stored energy of the reactive near-field region. In order to obtain a maximized antenna impedance bandwidth for an optimally small electrical size, in this paper, the volumetric efficiency of the antenna is augmented through the enhancement of patch thickness^33^.

The wavelength corresponding to the operating frequency (λ) of an antenna helps to determine its size or dimensions. Normally the wavelength for center frequency (λ_c_) is considered for determining the antenna size of narrowband antenna but the scenario is different for UWB/SWB antenna. Classical fundamental limitation theories are developed on the consideration of wavenumber for center frequency of operating band (k = 2π/λ_c_) of the antenna. Therefore, these theories are mostly applicable for the design of narrowband antenna since in this case, the wavelength difference between center and edge frequencies is quite less.

However, a close look at the wavelengths of the UWB/SWB antenna for different operating frequencies illustrates that the wavelength of center frequency is very much different from the lower and upper bound wavelengths. Hence, for UWB/SWB antennas, it is not feasible to use the concept of utilizing the central wavelength directly for the whole range of frequencies. Instead, a more appropriate approach is to define the antenna electrical size to satisfy the equation, k_L_*a* = 1. Here k_L_ represents the wavenumber corresponding to the lower bound of occupied bandwidth^33^. Table 2 presents the product (k_L_*a*) of this wavenumber *(k*_*L*_) and the antenna sphere radius (*a*) for all the designed and fabricated SWB antennas proposed in this work.

**Table 2:** Electrical size calculation of proposed SWB antennas.

Patch height (mm)	Lower frequency (F_L_) (GHz)	Wavelength at lower bound (λ_L_) (mm)	Wave number (k_L_)	Electrical size (k_L_$a$) (rad)
0.035	1.65 (simulated)	181.692	0.03458	1.037
28.5	1.39 (simulated)	215.678	0.02913	0.8739
0.035	1.75 (fabricated)	171.310	0.03667	1.1

### Impact of antenna volumetric efficiency on electrical size and bandwidth

Tables 2 and 3 represent the volumetric efficiency estimation in terms of the proposed antenna electrical size and bandwidth respectively. From Table 2, a comparative study between the simulated antenna cases depicts that the antenna electrical size (k_L_*a*), reduces from 1.037 to 0.8739 for increased patch height which is smaller than the theoretical limit (k*a* = 1). This suggests that by utilizing the enclosing spherical volume effectively, the antenna electrical size reaches a value lesser than unity and subsequently, exceeds the theoretical fundamental dimension limit of *ka* = 1. The fabricated thin patch antenna showed in Table 2 represents an electrical size of 1.1 which also indicate their proximity towards the fundamental limit.

**Table 3:** Proposed antenna performance in terms of bandwidth.

Substrate	Patch height (mm)	Impedance bandwidth (GHz)	Ratio bandwidth
RO 3003	0.035	1.65–160	96.96:1
RO 3003	28.5	1.39–160	115.10:1
RO 3003 (fabricated)	0.035	1.75–67	38.28:1

A high volumetric efficiency improves the impedance bandwidth of the antenna as well. Table 3 shows a bandwidth enhancement from 1.65–160 GHz to 1.39–160 GHz for a corresponding patch thickness increment from 0.035 mm to 28.5 mm. This may appear quite trivial at first glance; however, such a bandwidth enhancement in the lower frequency band allows the antenna to cover a range of new applications. In the end, this results in an improvement of ratio bandwidth with a value of as high as 115.10:1 for the antenna with thick patch. Considering the maximum measurement limit of 67 GHz, Table 3 depicts a ratio of 38.28:1 for the RO 3003 based fabricated antenna. These are by far the highest achieved bandwidth ratios amid all the proposed and practically implemented antennas reported in the literature.

The fundamental limit theory by Chu^31^ and Mclean^32^ illustrates that as the antenna size reduces, its quality factor, ${\text{Q}}$ gets increased. Antenna bandwidth and quality factor have a reciprocal relationship which implies a direct proportionality between bandwidth and antenna size. Therefore, in general, a reduction in the dimension of antenna incurs a decrement in its bandwidth. However, in this paper, the antenna patch height is increased to ensure an optimized volumetric efficiency which in turn maximizes the impedance bandwidth for an electrically small SWB antenna.

**Figure 14:**
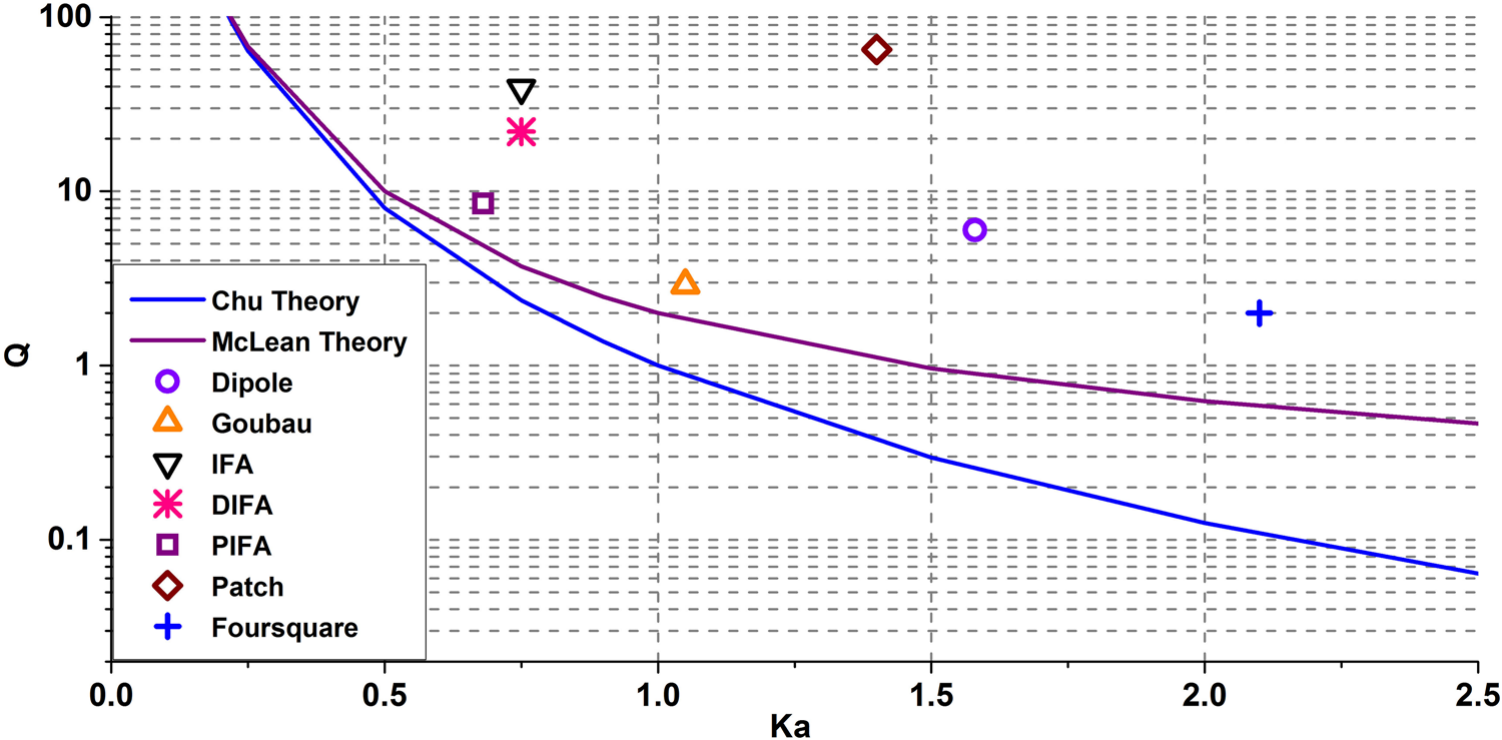
Comparison of several practical antenna (*Q, ka*) values to the fundamental limits curve^33^.

The list of antennas shown in Table 4 is obtained from literature in order to analyze the bandwidth-size relationship. Figure 14 shows the comparison of these practical antennas with the Chu and McLean fundamental limit curves. If the corresponding point of an antenna gets close to or falls onto any of the theoretical limit curves, the antenna is considered to have achieved the maximum bandwidth for its size. To achieve the electrically small size, the k*a* value of the antenna has to be less than or equal to 1. Figure 14 shows a wide variety of antenna types, however, none of them achieves the maximized bandwidth-size performance or gets close to the fundamental limits^33^.

**Table 4:** Characteristics of practical antennas used to analyze bandwidth-size relationships^33^.

Antenna	Center frequency (f_c_)	Bandwidth	Radius of enclosing sphere
λ/2 Dipole	300 MHz	36 MHz	λ/2
Goubau	300 MHz	75 MHz	0.166 λ
IFA	923.5 MHz	17 MHz	3.909 cm
DIFA	917 MHz	30 MHz	3.926 cm
PIFA	859 MHz	70 MHz	3.774 cm
λ/2 patch	3.03 GHz	32 MHz	2.237 cm
Foursquare	6 GHz	2.12 GHz	1.67 cm

**Figure 15:**
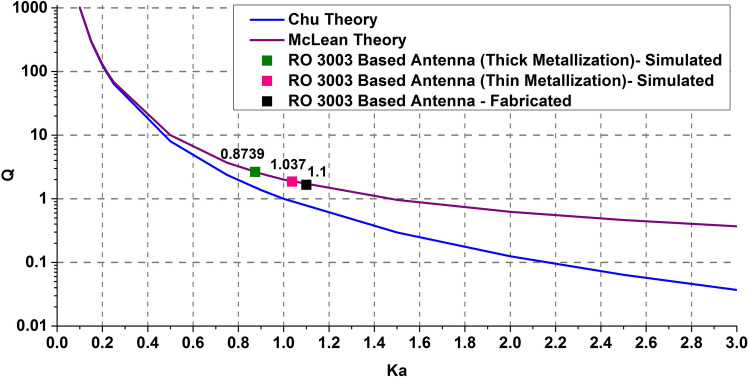
Comparison of proposed SWB antennas (simulated and fabricated) with the theoretical dimension limit curves.

In Fig. 15, the quality factors, ${\text{Q}}$ of antennas with designed (simulated) thin and thick metallic patch along with the fabricated antenna are plotted against their respective electrical size which are again compared with the theoretical fundamental dimension limit curves by Chu and Mclean. Here, Mclean’s exact expression is used to calculate the quality factor of the proposed antennas. It is evident that for the case of thick patch metallization, the antenna electrical size reaches quite close to Chu theory curve and marginally exceeds the Mclean curve^31,32^. Hence, it can be stated that the proposed super wide band antennas are designed to get very close to the fundamental limitation theory.

## Conclusions

This paper focuses on the design and experimental analysis of an electrically small super wide band antenna and its structural optimization to obtain proximity to the theory of classical fundamental dimension limit. A circular disc monopole based antenna is designed here with different patch heights and both of the respective antennas (with thin and thick patch) cover a much greater ratio bandwidth than 10:1 which indicates their operability as SWB antenna. It can be seen that the enhancement of antenna patch thickness results in a considerable improvement of ratio bandwidth. This illustrates that if the volumetric efficiency is augmented, the overall impedance bandwidth can be improved for a reduced antenna size. A comparative analysis with the classical Chu^31^ and Mclean^32^ theory demonstrates that all the proposed antennas get quite close to both the fundamental limit curves while the antenna with thick patch reaches the extent to surpass the Mclean curve. Thus, it can be deduced that the proposed thick patch-based antenna offers the maximized attainable bandwidth for the optimum compact size. The proposed antenna with thin patch is practically fabricated, and its experimental validation is carried out in order to indicate an excellent agreement between the simulated and measured results. These antennas operate in a varied range of popular wireless application bands including Wi-Fi, GPS, Bluetooth, UMTS, WiMAX, LTE, WLAN, PCS, UWB, satellite and terrestrial microwave communications. They can potentially be used as high-resolution sensors as well. These antennas can cover the recently approved IEEE 802.11aj (45 GHz band) standard^29^ for millimeter wave applications that can offer a maximum data rate of 14.17 Gbps. Combining this standard with the SWB technology would be an excellent breakthrough in the wireless communication research community.

## References

[1] Ke-Ren C, Sim C, Jeen-Sheen R (2011). A compact monopole antenna for super wideband applications. IEEE Antennas Wirel. Propag. Lett..

[2] Tran D, Lembrikov B (2010). On the design of a super wideband antenna. Ultra Wideband.

[3] Mahmud, S., Dey, S. & Saha, N. Super wide band wearable antenna: Assessment of the conformal characteristics in terms of impedance matching and radiation properties. In *IEEE International Symposium on Antennas and Propagation (ISAP)*, 563–566 (2012).

[4] Zhi Ning C, Kwai-Man L (2009). Antennas for Base Stations in Wireless Communications.

[5] Alomainy A, Hao Y, Pasveer F, Zhi Ning C (2007). Antennas for wearable devices. Antennas for Portable Devices.

[6] Volakis JL, Chen CC, Fujimoto K (2009). Small Antennas: Miniaturization Techniques & Applications.

[7] Bancroft R (2002). Fundamental Dimension Limits of Antennas.

[8] Kearney. D. *Small Antenna Options for Ultra-Wideband (UWB) Applications.*Masters Dissertation. Technological University Dublin (2009).

[9] Mahmud, M. S. & Dey, S. Design and performance analysis of a compact and conformal super wide band textile antenna for wearable body area applications. In *6th European Conference on Antennas and Propagation (EUCAP),*1–5. (2012).

[10] Xiao-Rong, Y., Shun-shi, Z. & Xue-Xia, Y. Compact printed monopole antenna with super-wideband. In *International Symposium on**Microwave, Antenna, Propagation and EMC Technologies for Wireless Communications*, 605–607 (2007).

[11] Wenjun, L. & Hongbo, Z. Super-wideband antipodal slot antenna. In *IEEE**Asia Pacific Microwave Conference (APMC),* 1894–1897 (2009).

[12] Barbarino S, Consoli F (2010). Study on super-wideband planar asymmetrical dipole antennas of circular shape. IEEE Trans. Antennas Propag..

[13] Jin XH, Huang XD, Cheng CH, Zhu L (2011). Super-wideband printed asymmetrical dipole antenna. Prog. Electromagn. Res. Lett..

[14] Tran, D. *et al.* A super wideband antenna. In *5th European Conference on**Antennas and Propagation (EUCAP),*2656–2660 (2011)

[15] Dorostkar MA, Islam MT, Azim R (2013). Design of a novel super wide band circular-hexagonal fractal antenna. Prog. Electromagn. Res..

[16] Almalkawi, M., Westrick, M. & Devabhaktuni, V. Compact super wideband monopole antenna with switchable dual band-notched characteristics. In *IEEE**Asia Pacific Microwave Conference (APMC),* 723–725 (2012).

[17] Bernety, H. M., Zakeri, B. & Gholami, R. A compact directional super-wideband antenna. In *21st Iranian Conference on**Electrical Engineering*(ICEE), 1–4 (2013)

[18] Liu J, Esselle KP, Hay SG, Zhong SS (2013). Compact super-wideband asymmetric monopole antenna with dual-branch feed for bandwidth enhancement. Electron. Lett..

[19] Dey, S. & Karmakar, N. C. Design of novel super wide band antennas close to the small antenna limitation theory. In *IEEE MTT-S International Microwave Symposium (IMS)*, 1–4 (2014)

[20] Aminudin Jamlos M (2019). Stacked stepped-fed super wideband antenna performance in free space and liquid medium for biomedical applications. IOP Conf. Ser. Mater. Sci. Eng.

[21] Manohar M, Kshetrimayum RS, Gogoi AK (2015). Super wideband antenna with single band suppression. Int. J. Microw. Wirel. Technol..

[22] Jianjun L, Esselle KP, Hay SG, Zhu S, Shunshi Z (2013). A compact super-wideband antenna pair with polarization diversity. IEEE Antennas Wirel. Propag. Lett..

[23] Jinger, R. & Agrawal, N. Design and development of compact super-wideband antenna with integrated bluetooth band. In *International Conference on Advanced Computing Networking and Informatics*, 381–387 (2019).

[24] Singhal S, Jaiverdhan, Singh AK (2019). Elliptical monopole based super wideband fractal antenna. Microw. Opt. Technol. Lett..

[25] Singhal S (2017). Asymmetrically fed octagonal Sierpinski band-notched super-wideband antenna. J. Comput. Electron..

[26] Rahman SU, Cao Q, Ullah H, Khalil H (2019). Compact design of trapezoid shape monopole antenna for SWB application. Microw. Opt. Technol. Lett..

[27] Balani W (2019). Design techniques of super-wideband antenna-existing and future prospective. IEEE Access..

[28] Q band. in Wikipedia, the free encyclopedia (2019). at https://en.wikipedia.org/wiki/Q_band. Accessed 25 November 2019.

[29] Haiming W, Wei H, Jixin C, Bo S, Xiaoming P (2014). IEEE (45GHz): a new very high throughput millimeter-wave WLAN system. China Commun..

[30] Harrington RF (1960). Effect of antenna size on gain, bandwidth, and efficiency. J. Res. Natl. Bureau Stand..

[31] Chu LJ (1948). Physical limitations of omni-directional antennas. J. Appl. Phys..

[32] McLean JS (1996). A re-examination of the fundamental limits on the radiation Q of electrically small antennas. IEEE Trans. Antennas Propag..

[33] Huynh, M.*Wideband compact antennas for wireless communication applications.*Ph.D. Dissertation. Virginia Polytechnic Institute and State University (2004).

[34] Yang, T., Davis W. A., & Stutzman, W. L. The design of ultra-wideband antennas with performance close to the fundamental limit. In *Proceedings of URSI General Assembly*, 234 (2008).

[35] Chen, X. & Massey, P. J. Operating Principles and Features of UWB Monopoles and Dipoles. In *IET Seminar on Ultra Wideband Systems, Technologies and Applications*, 131–152 (2006).

[36] Parizi SAR, Chattopadhyay S (2017). Bandwidth enhancement techniques. Trends in Research on Microstrip Antennas.

[37] Preradovic, S. *Chipless RFID system for barcode replacement*. Ph.D. Dissertation, Monash University (2009).

[38] Behdad N, Sarabandi K (2005). A compact antenna for ultrawide-band applications. IEEE Trans. Antennas Propag..

